# The Colour of the Hair

**Published:** 1912-01-27

**Authors:** J. S. Mackintosh

**Affiliations:** Corner House, Platt's Lane, Hampstead.


### THE COLOUR OF THE HAIR.

*To the Editor of* THE HOSPITAL.

SIR,—My attention has been called to an article in your columns dealing with certain observations I have made on the relative morbidity of "blondes" and "brunettes." In view of the results recently obtained by competent observers, there can be no doubt that in this country at the present day the brunettes have the advantage, but I hold strongly that their apparent superiority is relative, not absolute. By this I mean that the mere presence of a larger amount of pigment in the skin is not in itself sufficient to connote physical superiority over those less so endowed. Every established physical trait has a "survival value" in relation to the environment in which it first appeared, presumably by the action of natural selection. In view of the past history of the blonde "hordic" race, and the fact that it has provided the ruling caste to most European nations, it cannot be argued that it is essentially "inferior." The whole question may be thus formulated in regard to the individual. In what environment was evolved the race or stock he represents in his own person? To what degree do his present surroundings conform to, or differ from, the features of this environment? The blonde is at present hard pressed because economic forces are sweeping the population off the land into cities, and the blonde race, as Tacitus and other ancient writers have observed, never lived in cities but led a semi-nomadic life. For the same reason the gipsies, though deeply pigmented, have never taken to town-life, being essentially nomads. Close to my house, however, is a district where for generations they used to sojourn, and now that London has swallowed it up, some have attempted to cling to it, but with disastrous results, for they have shown themselves sadly liable to tubercle and other diseases when they substitute tenements for tents. It is true that intermediate types are commoner than typical blondes and brunettes, but the question of present *versus* past environment affects them also. Since the greater invasions of the country came to an end nearly a thousand years ago, and the country settled down to pastoral and agricultural pursuits, the formation of localised communities took place all over the country, and, undergoing in most cases little disturbance as century succeeded century, centreing themselves round some small market town or being isolated by the geographical conformation of the neighbourhood, these communities have become closely adapted to and specialised for local conditions. In the changes wrought by our advance from an agricultural to an industrial nation, we have, therefore, to deal not only with racial types but with sub-types changing their environment. I have been able to make some observations in this regard on domestic servants, shop assistants, etc., which I hope shortly to publish, and am glad to find that you agree with me that the subject is "worthy of careful and extended observations." Being a busy practitioner with a family to support, I am unable to give as much attention as I would like to the matter, but I hope to awaken the interest of others in these questions, and to see lines of research in this direction carried on which I am personally unable to pursue.

Believe me, yours faithfully,

J. S. MACKINTOSH, M.D., M.R.C.S., L.R.C.P.

Corner House, Platt's Lane, Hampstead.

January 24.

